# Machine Learning-Assisted Computational Screening of Metal-Organic Frameworks for Atmospheric Water Harvesting

**DOI:** 10.3390/nano12010159

**Published:** 2022-01-03

**Authors:** Lifeng Li, Zenan Shi, Hong Liang, Jie Liu, Zhiwei Qiao

**Affiliations:** 1 Guangzhou Key Laboratory for New Energy and Green Catalysis, School of Chemistry and Chemical Engineering, Guangzhou University, Guangzhou 510006, China; lilifeng@e.gzhu.edu.cn (L.L.); zenanshi@126.com (Z.S.); 2 Key Laboratory for Green Chemical Process of Ministry of Education, School of Chemical Engineering and Pharmacy, Wuhan Institute of Technology, Wuhan 430073, China

**Keywords:** metal-organic frameworks, water harvesting, molecular simulation, algorithm, absorption

## Abstract

Atmospheric water harvesting by strong adsorbents is a feasible method of solving the shortage of water resources, especially for arid regions. In this study, a machine learning (ML)-assisted high-throughput computational screening is employed to calculate the capture of H_2_O from N_2_ and O_2_ for 6013 computation-ready, experimental metal-organic frameworks (CoRE-MOFs) and 137,953 hypothetical MOFs (hMOFs). Through the univariate analysis of MOF structure-performance relationships, *Q*_st_ is shown to be a key descriptor. Moreover, three ML algorithms (random forest, gradient boosted regression trees, and neighbor component analysis (NCA)) are applied to hunt for the complicated interrelation between six descriptors and performance. After the optimizing strategy of grid search and five-fold cross-validation is performed, three ML can effectively build the predictive model for CoRE-MOFs, and the accuracy *R*^2^ of NCA can reach 0.97. In addition, based on the relative importance of the descriptors by ML, it can be quantitatively concluded that the *Q*_st_ is dominant in governing the capture of H_2_O. Besides, the NCA model trained by 6013 CoRE-MOFs can predict the selectivity of hMOFs with a *R*^2^ of 0.86, which is more universal than other models. Finally, 10 CoRE-MOFs and 10 hMOFs with high performance are identified. The computational screening and prediction of ML could provide guidance and inspiration for the development of materials for water harvesting in the atmosphere.

## 1. Induction

As we all know, 71% of the earth’s surface is covered by water, and the remaining 29% is land. At first glance, we have a lot of water resources; in fact, the water we use is mainly fresh water, and fresh water only accounts for 2.5% of all water resources on the earth [1]. Among them, approximately 69% of the fresh water is enclosed in the ice layer of Antarctica and Greenland, and the remaining 30% is stored in the ground, so the fresh water (such as river water and fresh water lakes) that humans can directly use accounts for only 0.4% of all water resources [1]. As population growth and living standards improve, water resources are becoming increasingly scarce, especially daily water for residents of arid regions. Currently, one third of the world’s population live in regions with medium and high water shortages. It is estimated that two thirds of the world will face water shortages by 2050 [2]. Therefore, the lack of fresh water has become one of the major crises to be resolved. At present, several technologies are being used to address this issue. Desalination is one of the main ways to develop new fresh water resources, but the construction of this infrastructure requires a lot of money and the production process is highly energy intensive [3]. In addition, since the main arid and water-scarce areas are far inland, there are never sea water resources available in these places. It is therefore especially important to develop a fresh water technology that can be used in arid areas.

The atmosphere of our planet contains a lot of water, which exists in the form of droplets and vapor. It accounts for about 10% of all fresh water [4]. Therefore, if atmospheric water can be efficiently harvested and used, water shortages can be greatly alleviated. At present, the two main methods for collecting water vapor from the air are vapor condensation and adsorption–desorption technology [5]. Condensation conditions are relatively harsh, usually requiring very high amounts of water vapor from the air (relative humidity, RH = 100%) and a large amount of energy, which is unrealistic when the relative humidity is less than 50% [4]. On the contrary, the adsorption–desorption technology can adsorb the water vapor from the air at low temperature, then, using low-grade energy such as natural sunlight or waste heat, it can desorb and condense it into liquid water. The entire process does not require additional energy. Obviously, adsorption–desorption technology is more convenient and energy-saving, and can be used in relatively low humidity regions.

The key to adsorption–desorption technology is to select a suitable adsorbent. Currently, the main adsorbents are polymers, zeolites, and silica gels. Unfortunately, these adsorbents have some shortcomings that cannot be ignored, such as low adsorption capacity and the fact that regeneration requires a lot of energy [6]. AMetal-organic frameworks (MOFs), a porous crystalline material, is self-assembled from metal ions and organic ligands [7]. Due to its characteristics of high porosity, large specific surface area and adjustable structure, it is regarded as a candidate for traditional adsorption. Although MOFs have long been used in the fields of gas adsorption [8], separation [9,10], storage [11,12], catalysis [13], and heat pumps [14,15], the use of MOFs to harvest water vapor from the atmosphere has only been proposed in recent years [1,16,17]. This is because structural stability of MOFs may deteriorate after absorbing water. As more MOFs with good water stability join the MOF family, the use of MOFs for atmospheric water harvesting has received widespread attention. Furukawa et al. [16] studied and evaluated the water adsorption properties of 23 materials including six newly synthesized zirconium-based MOFs and determined that MOF-841 can be used as a candidate for atmospheric water harvesting in arid regions. In 2017, Kim et al. [4] developed a heat pump system using the MOF as an adsorbent to collect water from the atmosphere. The principle is that the heat pump absorbs water at night, and uses low-grade energy such as natural sunlight to desorb and condense liquid water during the day. The system harvested 2.8 L of water per kilogram of MOF (MOF-801) per day at room temperature with relative humidity below 20%. Recently, Hanikel et al. [18] summarized the progress of atmospheric water harvesting and the use of MOF-designed water-collection equipment. Therefore, it is feasible to use MOFs to capture water vapor from the atmosphere. We need to screen out some super-hydrophilic MOFs in a huge MOF database. The water vapor in the atmosphere is extremely small compared to N_2_ and O_2_, so we need super-hydrophilic and highly selective MOFs to efficiently capture water from the atmosphere. It is very difficult to select suitable MOFs from so huge a database by experimental verification. 

The emergence of high-throughput computational screening (HTCS) provides a possibility for solving the problem. Qiao et al. [19] used HTCS to select MOFs suitable for separating CO_2_/N_2_ and CO_2_/CH_4_ from 4764 computation-ready, experimental MOF (CoRE-MOF) databases. In fact, HTCS includes Monte Carlo simulations (MC) and machine learning (ML) [20], and previous studies on HTCS have usually relied on molecular simulation. In recent years, ML has been widely applied to various fields, including image recognition [21], natural language processing [22], data classification and mining [23,24], and material performance prediction [25]. In our previous work, Shi et al. [26] combined MS and ML to screen MOFs with good performance that can be used for adsorption heat pumps. In addition, according to two ML methods with good prediction effects, we determined that the heat of adsorption is the key descriptor that determines the performance of the heat pump. In the work of Dureckova et al. [27], a gradient boosting regression tree (GBRT) model was applied to predict the CO_2_ working capacity and CO_2_/H_2_ adsorption selectivity of carbon capture. It was found that the *R*^2^ values of predictive CO_2_ working capacity and CO_2_/H_2_ selectivity were 0.944 and 0.872, respectively. Hypothetical MOFs (hMOFs) can be automatically generated by different metals, linkers. and topologies in computer software. Wilmer et al. [28] generated 137,953 hMOFs from a library of 102 building blocks and screened 300 hMOFs with a higher capacity for methane storage than known CoRE-MOFs. Wu et al. [29] formed a new data set with 130,397 hMOFs and 37 feature descriptors including Henry’s coefficient, atomic number density, and functional group number density. They found that the hMOFs with optimal methane-storage capacities exhibit *ϕ* of 0.65–0.88, VSA of ~2250 m^2^·cm^–3^, etc.

The combination of machine learning and molecular simulation of HTCS has greatly increased the speed of discovering new materials [27,30], because ML suitable for specific systems will reduce the number of simulated materials, especially for the updated database of material. Recently, Pardakhti et al. [31] used the trained random forest (RF) of the ML model to predict the methane adsorption of ~130,000 hMOFs. The results showed that the speed of ML was several orders of magnitude faster than traditional MS. The combination of MS and ML has developed into the current main method of screening materials. In Shi et al.’s review [7], several ML methods were considered to possess better prediction performance, such as back propagation neural network and random forest. Therefore, in this work, we selected these methods, as well as gradient boosting regression tree and neighbor component analysis, on the basis that they have good predictive performance for water harvesting on MOFs.

In the present work, we apply MC and three ML models to study the performance of water harvesting on MOFs. Based on the established structure-performance relationship, all three types of machine learning achieve a relatively good predictive effect. Then we obtained the main descriptors that played an important role in the performance of MOFs for the capture of water, and finally obtained super hydrophilic MOFs. This may provide guidance for experimental workers to synthesize available MOFs.

## 2. Models and Methods

### 2.1. Molecular Models

The crystal structures of the version 2017 of 6013 CoRE-MOFs were collected and established by Chung et al. [32,33] removing the free and coordinated solvent molecules. A large crystallographic dataset of 137,953 hMOFs was designed by Wilmer et al. [28] using 102 building blocks and six different topologies. Five structural descriptors including the largest cavity diameter (LCD), pore-limiting diameter (PLD), volumetric surface area (VSA), void fraction (*ϕ*), density (*ρ*), and an energy descriptor of heat of adsorption (*Q*_st_), were used to quantitatively describe the structure of the MOF. The reasons for the selection of these six descriptors are as follows: (1) they possessed the strong structure-performance relationships between gas and MOFs, confirmed by many previous works [28,32,34,35] of high-throughput calculation of MOFs, which means that these six MOF descriptors have a greater possibility of achieving the accuracy prediction in ML models than the thousands of other descriptors that could have been used; (2) these six descriptors could be applied in accuracy prediction of ML, which coincide with many ML works [36,37,38,39]; (3) these descriptors are relatively easy to measure in the experiment, and they can be used directly to guide the synthesis and application of MOF. The LCD and PLD in each CoRE-MOF were estimated using Zeo++ [40]. The VSA and *ϕ* were determined using the diameter of 0.364 nm and 0.258 nm of N_2_ and He as a probe under the RASPA package, respectively [41]. The *Q*_st_ was calculated by the *NVT*-Monte Carlo (MC) with the Widom method in RASPA under infinite dilution conditions, where *N*, *V*, and *T* are the number of particles, the volume of system, and the temperature of the system, respectively [41]. 

The partial atomic charges of MOFs were rapidly estimated and evaluated using the new MEPO-Qeq [42] method trained to reproduce density function theory (DFT), the extended electrostatic potential fitted charges using the Repeating Electrostatic Potential Extracted Atomic (REPEAT) method [43]. The LJ potential parameters of all CoRE-MOFs were obtained from the universal force field (UFF) [44], as listed in Table S1. In our previous work, it was shown that combining the force fields and MEPO-Qeq method can accurately and quickly predict the adsorption and capture of gases in various MOFs [26,45]. The force field parameters of N_2_ and O_2_ molecules were described by the transferable potentials for phase equilibria (TraPPE) force field [46], as demonstrated in Table S2. The TIP4P-Ew [47] model was used to simulate H_2_O molecules with LJ sites on the O and H atoms, along with the partial charges on H atoms and a dummy atom. A three-site model was applied to mimic a CO_2_ molecule, which has a C-O bond length of 0.116 nm and a bond angle ∠OCO of 180° [48]. Similarly, an N_2_ molecule was modeled as a three-site model with the N-N bond length of 0.110 nm.

### 2.2. Monte Carlo Simulations

To capture water from the air, the Henry’s constants of H_2_O, N_2_, and O_2_ were calculated at 298 K using the Widom particle insertion method [49], and then the selectivity *S*_0[H_2_O/(N_2_+O_2_)]_ was calculated by the Henry’s constant of three gas molecules. In this study, the MOF with the larger Henry’s constant of H_2_O (*K*_H2O_) and higher *S*_0[H_2_O/(N_2_+O_2_)]_ is regarded as excellent candidate. Notice that Henry’s constant of water is calculated based on the interaction of a water molecule with the framework, which is mainly designed to simulate the extremely low water-molecule content in extreme environments such as deserts (it can be regarded as only one molecule of water in the air). It is noteworthy that, although grand canonical MC (GCMC) is an accurate estimation for the adsorption performance of MOFs, it is difficult to accurately calculate the adsorption loading of H_2_O. This is because although the structure of a water molecule is very simple, a molecular H-O-H hydrogen bond angle and dipole moment can change continuously during the adsorption process, which further complicates the adsorption [50]. Thus, the H_2_O adsorption isotherm in most adsorbing has a jump in a narrow range of vapor pressure. This jump is very difficult to calculate during the GCMC simulation. Currently, there was still not a suitable force field or H_2_O model, which could be used to screen the adsorption loading of H_2_O in most CoRE-MOFs by GCMC. After the GCMC simulation was repeatedly tested, only several MOFs could be accurately predicted with a relatively good level of agreement with the experimental isotherm [46,50,51,52] Therefore, for a large scale of screening of CoRE-MOFs, the *K*_i_ was used to calculate the adsorption selectivity of H_2_O in this work. For further explanation, see the supporting information (SI).

The simulation unit cell extended to at least 2.4 nm along each dimension, and periodic boundary conditions were applied in the three dimensions. It was assumed that the framework atoms of MOFs were rigid and fixed during the simulations. To calculate the LJ interaction, the long-range corrected spherical cut-off radius was set to 1.2 nm. The Ewald summation [53] method was used to estimate the electrostatic interaction between the frameworks and gas molecules as well as between the gas molecules. The number of MC cycles was 100,000; the first 50,000 cycles were performed to the simulation of the equilibrium system, and the last 50,000 cycles were run for ensemble averages. After testing, it was shown that the effect of increasing the MC cycle on the adsorption results was negligible. All simulations were carried out under the RASPA package [41].

### 2.3. Machine Learning Method

To find out which of the machine-learning (ML) models is suitable for predicting the relationship between the six descriptors (LCD, *ϕ*, VSA, PLD, *ρ*, *Q*_st_) and the selectivity *S*_0[H_2_O/(N_2_+O_2_)]_ of MOFs, further information was sought by ML models. The three kinds of ML employed for the prediction of *S*_0[H_2_O/(N_2_+O_2_)]_ were random forest (RF), gradient boosting regression tree (GBRT), and neighbor component analysis (NCA), which were run in Statistics and Machine Toolbox Learning under Matlab2019a software. Because the magnitude of the selectivity data span was very large and cannot be predicted directly, it needed to be pre-processed first; that is, the value of *S*_0[H_2_O/(N_2_+O_2_)]_ was taken by the logarithm (log_10_(*S*_0[H_2_O/(N_2_+O_2_)]_)) to narrow the enormous difference in the various data. In this work, after the five-fold cross-validation evaluated all possible values of each parameter, three ML algorithms programmatically selected the optimal parameter values for the final calculation and prediction, in which the six descriptors were regarded as the input variable and log_10_(*S*_0[H_2_O/(N_2_+O_2_)]_) as output variable of ML. The key parameters were optimized by five-fold cross-validation and grid-search, as listed in Table S3. All data were divided into five folds. For each cycle, four-fifths of the data were selected randomly as a training set, and one-fifth of the data as a test set. The ML model was run five times for each group value of key parameters by the five-fold cross-validation. The average determinate coefficient (*R^2^*) of test sets in five-fold cross-validation was adopted to indicate the performance of the model built by different parameter groups.

$$R^{2}=1-\frac{\sum_{i=1}^{n}{\left(y_{i}-f_{i}\right)}^{2}}{\sum_{i=1}^{n}{\left(y_{i}-{\bar{f}}_{i}\right)}^{2}}$$

where *n*, *y_i_*, *f_i_*, and $\overset{–}{f_{i}}$ refer to the number of MOFs, simulated value, ML predicted value, and average ML predicted value, respectively.

In view of the maximum average *R*^2^, the optimal parameters could be automatically obtained by the strategy of parameter optimization. Except the optimized parameters, the other parameters were the default values, as listed in Table S4. Secondly, the entire data set of 6013 CoRE-MOFs was adopted to train the model with optimal parameter values. Finally, the data of 10,000 hMOFs were tested.

Among them, NCA [54,55] is a supervised learning algorithm that learns the feature weights using a diagonal adaptation. RF is made of multiple decision trees to achieve comparatively higher robustness, and its output is the average of the prediction results of multiple trees [31]. Similarly, GBRT is also an aggregation method by decision tree, which creates the optimal split criterion by continuously minimizing the least squares-regression error for the reduction of computing residual last time. More details of the three MLs are listed in the SI.

## 3. Results and Discussion

### 3.1. Univariate Analysis

**Figure 1:**
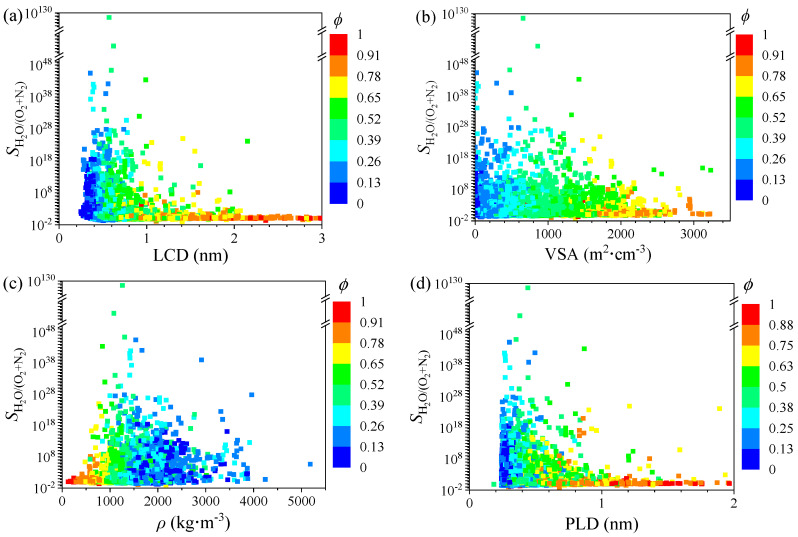
The relationship of selectivity *S*_0[H_2_O/(N_2_+O_2_)]_ *versus* (**a**) LCD, (**b**) VSA, (**c**) *ρ*, and (**d**) PLD. The color code represents void fraction *ϕ*.

To explore the effect of the six MOF descriptors on water harvesting performance, we used univariate analysis to understand the relationship between each descriptor (LCD, *ϕ*, VSA, *ρ*, PLD, and *Q*_st_) and the selectivity *S*_0[H_2_O/(N_2_+O_2_)]_. In Figure 1, the scale of *S*_0[H_2_O/(N_2_+O_2_)]_ is very large, because the adsorption behavior of vapor water is very special; it is different from most gases. It is a typical multilayer adsorption. The adsorption of vapor water in MOFs can be divided into two stages. Firstly, based on the interaction between vapor water and MOFs, the water molecules are gradually adsorbed in the pore wall of MOFs. Second, with the increasing of water molecules entering into the framework, strong hydrogen bonds are formed between water and water molecules, leading to remarkable multilayer adsorption. Thus, it is extremely important in the adsorption process of vapor water that the first layer of water is successfully adsorbed in MOFs. Therefore, the difference in selectivity of H_2_O between hydrophilic and hydrophobic MOFs is extremely large, leading to the data with very high value. In addition, the content of water vapor in the atmosphere is very small, especially in desert areas. The V-shaped adsorption isotherm and very high selectivity of water could be helpful to achieve the capture of H_2_O in these extreme environments. Figure 1a shows the relationship of the *S*_0[H_2_O/(N_2_+O_2_)]_ and LCD. The selectivity is close to 0 in the range of LCD less than 0.27 nm, which may be because the molecules of H_2_O with the dynamic diameter of 0.264 nm cannot enter the pores of the MOF. As the LCD continues to increase, the *S*_0[H_2_O/(N_2_+O_2_)]_ gradually decreases and eventually stabilizes at less than 1 (approximately 0.01). The *S*_0[H_2_O/(N_2_+O_2_)]_ is less than 1, indicating that the MOF does not have the ability to selectively adsorb H_2_O vapor, but preferentially adsorbs N_2_ and O_2_ in the atmosphere. This process reflects the change from shape selective to inverse-shape selective adsorption. The relationship of the *S*_0[H_2_O/(N_2_+O_2_)]_ and VSA is shown in Figure 1b. In the region where VSA is close to 0, it shows a higher selectivity. When VSA continues to increase, the selectivity reaches its highest point, then gradually decreases. This is because when the VSA is small, the pores of the MOF can accommodate H_2_O molecules, and when the VSA is too large, the accessible surface of all molecules in the MOF increases, so the contact probability of the N_2_ and O_2_ with their optimal adsorption sites increases. Therefore, the selectivity will decrease; that is, the selective separation of H_2_O vapor cannot be achieved. The super hydrophilic MOFs with high selectivity and high Henry’s constants have a VSA of less than 1000 m^2^·cm^−3^, except that the VSA of HUZSUR01 is 1422.66 m^2^·cm^−3^.

Figure 1c shows the relationship of the *S*_0[H_2_O/(N_2_+O_2_)]_ and density *ρ*. Density and void fraction are correlated descriptors. It is worth noting that as the selectivity increases, the density and void fraction change in opposite directions. This is not difficult to understand. The larger the porosity, the larger the pore volume of the MOF and the lower its density. When the *ρ* is less than 1260 kg·m^−3^, the *S*_0[H_2_O/(N_2_+O_2_)]_ increases as the density increases, and then the selectivity decreases with the increase of the *ρ*. In Figure 1, the void fraction *ϕ* is mapped in the subplots as color codes. The MOFs with high selectivity have a medium-range of *ϕ* (0.20–0.62), except for MOF HEWFUL (*ϕ* = 0.16). This is because too large and too small pores are not suitable for selective separation. The pore is too small to prevent the molecule of H_2_O from entering, thus hindering the adsorption. Conversely, if the pore is too large, the interaction between the adsorbed molecules and the MOF will be weakened, which is not conducive to selective separation. The *S*_0[H_2_O/(N_2_+O_2_)]_ *versus* PLD is shown in Figure 1d. This trend is similar to the relationship of the *S*_0[H_2_O/(N_2_+O_2_)]_ and LCD. The highest *S*_0[H_2_O/(N_2_+O_2_)]_ are observed at PLD with 0.4 nm, which is approximately equaled to the kinetic diameter of N_2_ and O_2_ (0.364 nm and 0.346 nm, respectively). Although the PLD of some MOFs is smaller than the dynamic diameter of H_2_O, it is still possible for water molecules to enter these MOF channels. On the one hand, MOF pores are not regular, H_2_O molecules may enter from other larger pores; on the other hand, the kinetic diameter of H_2_O is estimated by empirical estimation, which is usually larger than the actual size [56], so the molecule of adsorbate may be adsorbed in the MOF.

**Figure 2:**
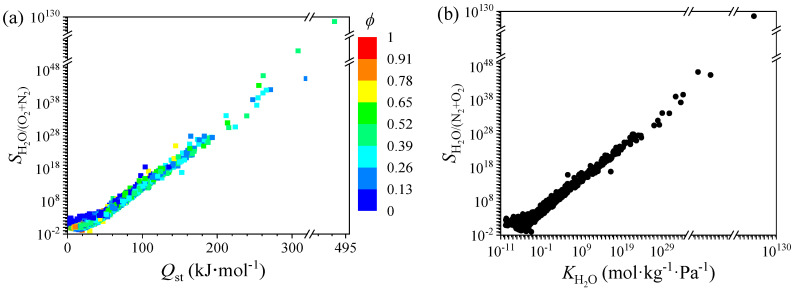
The relationship of selectivity *S*_0[H_2_O/(N_2_+O_2_)]_ *versus* (**a**) *Q*_st_, (**b**) *K*_H2O_. The color code represents void fraction *ϕ*.

Figure 2a shows that the selectivity increases with the heat of adsorption, which shows a monotonic upward trend. The trend is almost linear, indicating that the isosteric heat of adsorption and the selectivity are strongly correlated variables. When the range of *Q*_st_ is 270–480 kJ·mol^−1^, the MOF has its highest *S*_0[H_2_O/(N_2_+O_2_)]_. Since we simulated the adsorption of a single H_2_O molecule in MOF at infinite dilution, so the heat of adsorption can characterize the strength of the adsorption. Therefore, *Q*_st_ may be a key descriptor for determining *S*_0[H_2_O/(N_2_+O_2_)]_. This phenomenon also appeared in the CO_2_ [57] adsorption and thiol capture [45] from the air in our previous works. Figure 2b plots the relationship of the *S*_0[H_2_O/(N_2_+O_2_)]_ and the Henry’s constants of water *K*_H2O_ [58]. On a logarithmic scale, the scatter plot of the *S*_0[H_2_O/(N_2_+O_2_)]_ and the *K*_H2O_ shows an upward trend. The Henry’s constant is a parameter that measures the affinity between the optimal adsorption site of the adsorbent and the adsorbate. The larger Henry’s constant indicates that the interaction between adsorbent and adsorbate molecule is stronger, making adsorption-based separation achievable. Thus, it is necessary that the MOF with large *K*_H2O_ is required to harvest H_2_O vapor from the air in arid areas (RH ≈ 20%). From Figure 1 and Figure 2, the *Q*_st_ seems to be the most important descriptor, and its relationship with selectivity is the most obvious. After linear, binomial, and trinomial fitting for *S*_0[H_2_O/(N_2_+O_2_)]_~*Q*_st_, the *R*^2^ of binomial fitting could achieve 0.97 and remain stable by using the trinomial fitting, as is shown in Figure S2. The deviation of two points with highest *S* in the linear fitting makes a relatively lower *R*^2^ than both the binomial and trinomial fitting. In fact, the *R*^2^ for linear fitting is only 0.93, and it has no accuracy prediction for data in the range of log *S* > 47. In view of the fitting, we can simply estimate and understand the structure-property relationships of MOFs for the atmospheric water harvesting. Therefore, *Q*_st_ is very worthy of attention during the screening process.

### 3.2. Machine Learning

**Figure 3:**
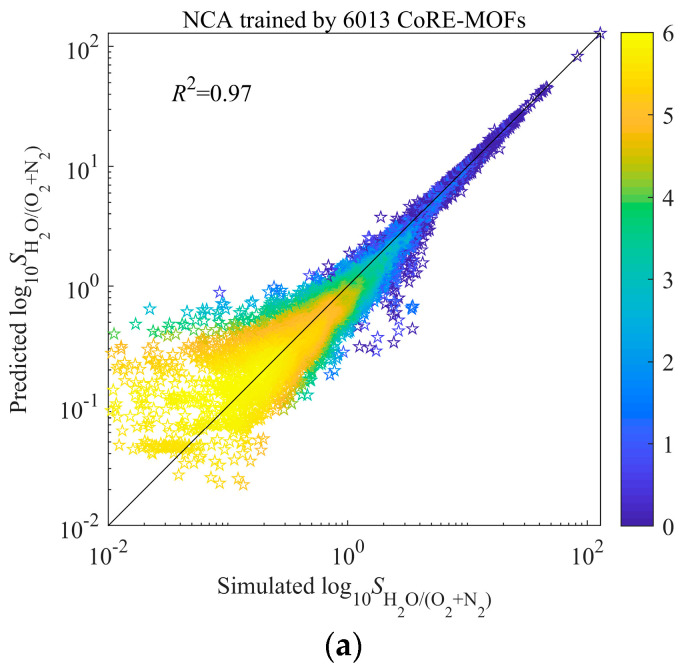

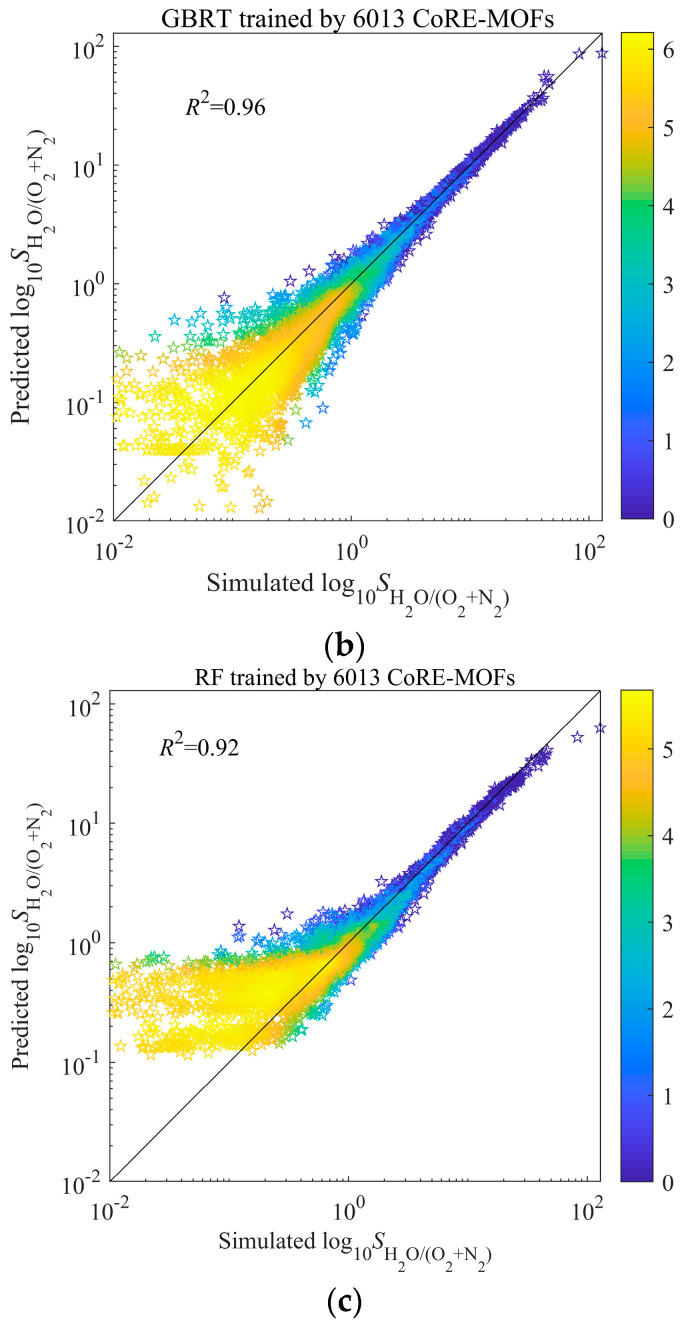
Model (**a**) NCA, (**b**) GBRT, and (**c**) RF trained by 6013 CoRE-MOFs. The color represents a base-*e* logarithm of the number of MOFs.

At present, ML has been widely used to predict the performance of materials. Through univariate analysis, only the influence of a single descriptor can be obtained, and ML can not only predict the relationship of structure-performance, but also obtain the common impact of multiple descriptors on performance. In our study, the optimal parameters were obtained by five-fold cross-validation and grid-search. The average *R*^2^ of test sets in five-fold cross-validation was adopted to indicate the performance of the model built by different parameter groups. The final model trained by all 6013 pieces of data and optimal parameters. The results are showed in Figure 3a–c. The order of three ML is NCA > GBRT > RF. Compared to GBRT, NCA performs better in the range of high log_10_(*S*_0[H_2_O/(N_2_+O_2_)]_), while compared to RF, NCA performs better in the range of both low and high log_10_(*S*_0[H_2_O/(N_2_+O_2_)]_). The reason for this may be the different learning styles of the model, such as feature learning.

**Figure 4:**
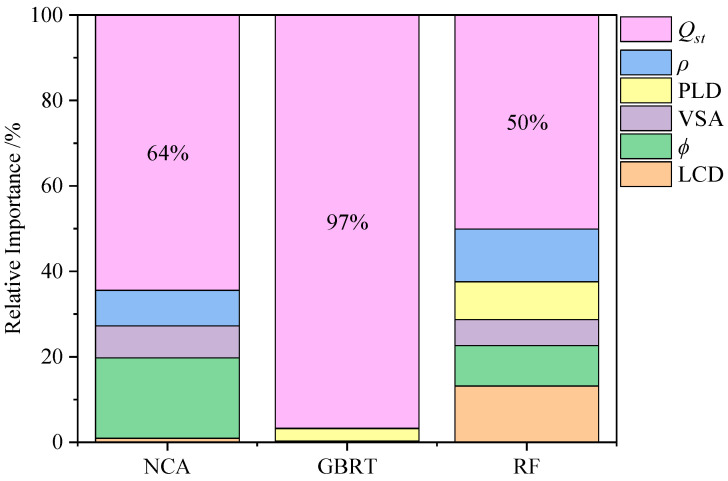
Relative importance of the six descriptors on the selectivity of MOFs by three ML prediction.

To further understand the relative importance of the six descriptors for the *S*_0[H_2_O/(N_2_+O_2_)]_, we calculated the weight of each descriptor by three MLs. The weight of the descriptors was calculated while the model was being constructed. The value of relative importance was computed by the normalization of the weight of the six descriptors, as shown in Figure 4 and Table S5. Due to the different characteristics of models, ML shows the relative importance of the descriptors in different ways. However, they all have a point of comparison, which is that the proportion of *Q*_st_ is more than 50%, especially for GBRT almost only built by a variable (*Q*_st_). The order of the six descriptors is *Q*_st_ > *ϕ* > *ρ* > LCD ≈ VSA > PLD. *Q*_st_ seems to govern the MOF performance in this work, because the concentration of vapor water in air is close to the condition of infinite dilution. The result shows that *Q*_st_ holds an absolute advantage importance relative to others, as in Section 3.1, which provides a guide for designing the best MOFs of adsorption of water vapor in the experiment. 

**Figure 5:**
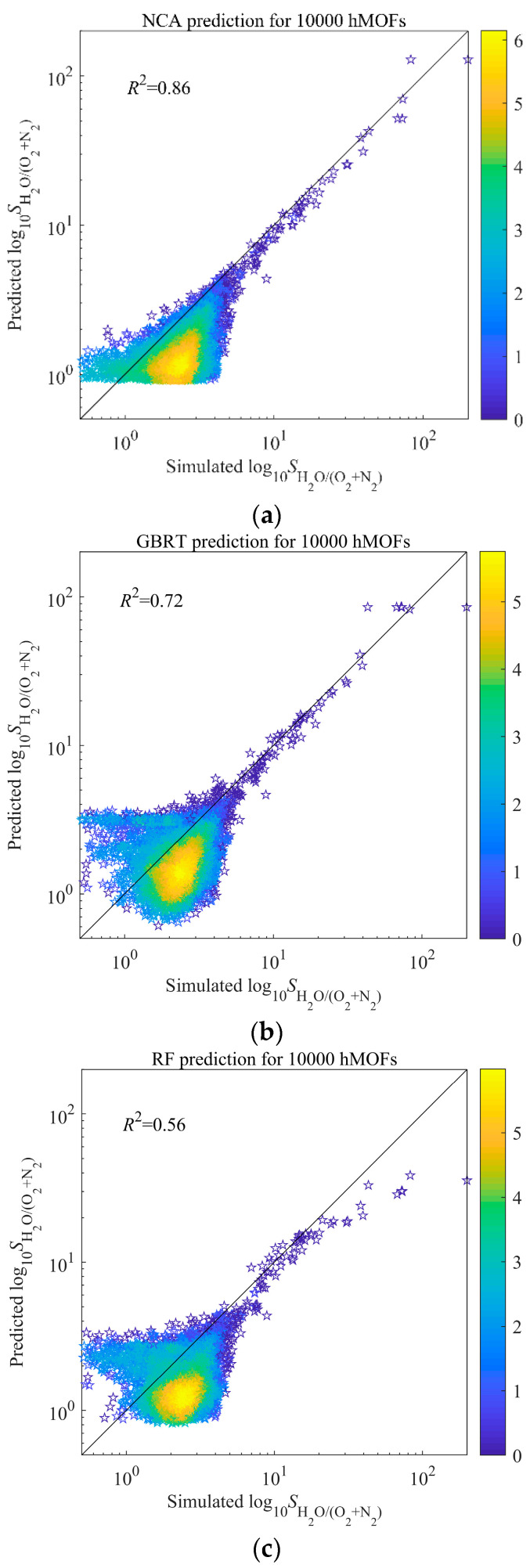
Model (**a**) NCA, (**b**) GBRT, and (**c**) RF prediction for 10,000 hMOFs. The color represents a base-*e* logarithm of the number of MOFs.

Furthermore, the predictive ML model should be used to accelerate the new HTCS for the other MOF database. Of course, both the simple binomial/trinomial fitting and ML model could achieve this H_2_O-MOF system, because of the strong relativity of *Q*_st_, but ML model would possess higher universality for the other gas-MOF system. Thus, we have added the prediction of a new MOF database (137,953 hMOFs) [28] by ML model, which was trained by 6013 CoRE-MOF datasets. First, *Q*_st_ was calculated for all 137,953 hMOFs, and then we selected 10,000 hMOFs with the highest *Q*_st_ for the new prediction, because *Q*_st_ has the highest importance. As shown in Figure 5a–c, after the predicted results were compared with simulated results by molecular simulation, *R*^2^ of the prediction in NCA could reach 0.86. The reasons for the differences of performance between training and predicting are that there exist some differences between the CoRE-MOF and hMOF databases. For examples, there are more than 350 topologies in the CoRE-MOFs database, while there are only six topologies in the hMOFs database, which leads to a diversity gap in those databases; CoRE-MOFs contain much more open metal sites or non-skeleton ions than hMOFs [28]. In this work, the establishment and evaluation of models are finished by 6013 CoRE-MOFs. Ten thousand hMOFs are the extra data, which are different from CoRE-MOFs in some aspects and do not participate in the establishment and evaluation of models. The difference between NCA and GBRT/RF could be that GBRT overemphasizes the importance of *Q*_st_ (relative importance ≈ 97% in Figure 4); that is, the GBRT model is almost only built by a variable (*Q*_st_) and RF may fail to grasp the importance of features other than *Q*_st_. Therefore, GBRT and RF may be suitable for the prediction of CoRE-MOFs but not hMOFs, which also means NCA is more universal. Nevertheless, the prediction of NCA for 10,000 hMOFs still shows the sufficient predictive ability of the model, but it is usually not as effective as the original dataset [59]. Moreover, it can be found that, when a hMOF possesses high selectivity (log_10_*S* > 5.3), the model performs very well. Thus, the ML model obtained by the CoRE-MOF database can pre-screen out low-performance MOFs to greatly reduce the running time of molecular simulation. Based on the ML algorithm, 80 hMOFs with high performance (log_10_*S* > 5.3) could be precisely screened out, and then only the selected 80 hMOFs would have to have their simulated adsorption behavior calculated, as opposed to 137,953 hMOFs, saving a considerable amount of time and computing resource. Finally, the optimal hMOFs were listed in Table S6.

## 4. Best CoRE-MOFs 

According to the principle that both the Henry’s constants of H_2_O and the selectivity of excellent MOFs are large, we selected 10 optimal CoRE-MOFs for harvesting water from the air based on the order of the selectivity of MOFs from high to low, as listed in Table 1. Among them, the best MOF is QUTHAP, whose *K*_H2O_ and *S*_0[H_2_O/(N_2_+O_2_)]_ are 2.78 × 10^124^ and 4.14 × 10^128^, respectively. The range of LCD, *ϕ*, VSA, PLD, *ρ*, and *Q*_st_ of 10 MOFs is 0.035–0.988 nm, 0.16–0.62, 10.46–1422.66 m^2^·cm^−3^, 0.264–0.867 nm, 842.16–2912.23 kg·m^−3^, and 261.01–479.91 kJ·mol^−1^, respectively. The range of *Q*_st_, VSA, and *ϕ* shows significant agreement with the analysis of Section 3, while the others show less because the relationships between them and selectivity are less obvious. Obviously, the Henry’s constants and selectivity show a proportional trend, which is consistent with our univariate analysis. Moreover, we give more excellent hydrophilic MOFs for the further test. The top 200 CoRE-MOFs and top 200 hMOFs were listed in the Excel file of SI.

## 5. Conclusions

In summary, we simulated the adsorption behaviors of H_2_O, N_2,_ and O_2_ on 6013 CoRE-MOFs and 137,953 hMOFs by HTCS and ML. Then, after the relationships between selectivity and six MOF descriptors (LCD, *ϕ*, VSA, *ρ*, PLD and *Q*_st_) were analyzed, respectively, *Q*_st_ of H_2_O was shown to possess a strong correlation with the MOF ability for the capture of H_2_O. Furthermore, three ML algorithms were employed to predict the adsorption performance for each CoRE-MOF, indicating that NCA with a five-fold cross-validation accuracy of *R*^2^ = 0.97 is the best algorithm for the prediction of selectivity and that the rank of their predictive ability is NCA > GBRT> RF. Continuously, the relative importance of the six descriptors by MLs could demonstrate that the *Q*_st_ took the absolute predominance for designing MOFs with optimal selectivity of H_2_O/air. In addition, from the three models applied to predict the selectivity of hMOFs, it was found that the predicted *R*^2^ of NCA can reach 0.86; NCA is more universal for gas-MOFs systems than other models. Finally, the ten MOFs with the best performance were screened out by the statistical methods. They were potential candidates for the capture of H_2_O from air, especially for QUTHAP. The bottom-up microscopic insights obtained from this study offer experimentalists the guidelines for the development of MOFs with high performance for atmospheric water harvesting.

## Figures and Tables

**Table 1:** Top 10 CoRE-MOFs with optimal performance of water harvesting.

No.	CSD Code *^a^*	LCD (nm)	*ϕ*	VSA (m^2^·cm^−^^3^)	PLD (nm)	*ρ* (kg·m^−3^)	*Q*_st_ (kJ·mol^−1^)	*K*_H2O_ (mol·kg^−1^·Pa^−1^)	*S*_0[H2O/(N2+O2)]_
**1**	QUTHAP	0.569	0.44	654.97	0.441	1257.79	479.91 ± 8.31	2.78 × 10^124^	4.14 × 10^128^
**2**	CAJWIV	0.620	0.49	856.37	0.380	1078.91	307.79 ± 10.19	4.30 × 10^77^	6.85 × 10^82^
**3**	PIBLUJ	0.595	0.39	473.39	0.352	1304.13	261.19 ± 5.98	5.81 × 10^41^	2.35 × 10^46^
**4**	LIRVAK	0.355	0.22	17.88	0.301	1535.50	318.82 ± 7.42	7.16 × 10^44^	3.11 × 10^45^
**5**	HUZSUR01	0.988	0.62	1422.66	0.867	842.16	255.45 ± 8.23	3.18 × 10^39^	2.48 × 10^43^
**6**	HEWFUL	0.558	0.16	293.03	0.494	1665.78	271.03 ± 3.11	1.63 × 10^36^	1.31 × 10^42^
**7**	YUJWAD	0.388	0.26	16.62	0.264	1429.41	265.55 ± 9.25	6.35 × 10^36^	9.73 × 10^41^
**8**	YUJWAD01	0.398	0.28	42.73	0.270	1409.42	261.01 ± 9.77	1.73 × 10^36^	1.30 × 10^41^
**9**	- *^b^*	0.384	0.26	10.46	0.271	1414.41	254.38 ± 9.03	1.85 × 10^34^	4.26 × 10^39^
**10**	ECUFEP	0.532	0.22	491.72	0.447	2912.23	247.36 ± 7.13	2.53 × 10^32^	1.17 × 10^39^

*^a^* CSD code is the number of MOFs in the Cambridge Structural Database. *^b^* This MOF came from Tominaka et al.’s work [60].

## Data Availability

Not applicable.

## Supplementary Materials

The following supporting information can be downloaded at: https://www.mdpi.com/article/10.3390/nano12010159/s1, Table S1: Lennard-Jones parameters of MOFs; Figure S1: Models of N_2_ and O_2_; Table S2: Lennard-Jones parameters and charges of adsorbates; Explanation about calculation approach; Figure S2: Linear, binomial and trinomial fitting; Figures S3–S5: Details of three ML algorithms; Table S3: Type and the range of key parameters in the optimization; Table S4: Parameters of 4 ML; Table S5: Predictive importance of six descriptor by four ML algorithms; Table S6: Details of top ten hMOFs with optimal performance of water harvesting; Excel file: Top 200 CoRE-MOFs and hMOFs.

## Nomenclature

LCD	largest cavity diameter, nm
PLD	pore-limiting diameter, nm
VSA	volumetric surface area, m^2^·cm^−3^
*ϕ*	void fraction
*ρ*	density, kg·m^−3^
*Q*_st_	an energy descriptor of heat of adsorption, kJ·mol^−1^
*K*_H_2_O_	Henry’s constant of H_2_O, mol·kg^−1^·Pa^−1^
*S*_0[H_2_O/(N_2_+O_2_)]_	The initial selectivity of water molecules relative to nitrogen and oxygen adsorbed by MOFs.
