# Supplementary material for: Machine Learning-Assisted Computational Screening of Metal-Organic Frameworks for Atmospheric Water Harvesting
Source: Nanomaterials (Basel). 2022 Jan 3;12(1):159. doi: 10.3390/nano12010159 (PMC8746952; doi:10.3390/nano12010159)
Supplement: Supplementary file 1 [file nanomaterials-12-00159-s001.zip › Supplementary Materials.pdf]

Supporting Information

Machine Learning-Assisted Computational Screening of Metal-organic Frameworks for Atmospheric Water Harvesting

Lifeng Li [1,†], Zenan Shi [1,†], Hong Liang [1,*], Jie Liu [2,*] and Zhiwei Qiao [1,*]

1 Guangzhou Key Laboratory for New Energy and Green Catalysis, School of Chemistry and Chemical
Engineering, Guangzhou University, Guangzhou, 510006, China; lilifeng@e.gzhu.edu.cn (L.L.); zenanshi@126.com (Z.S.)

2 Key Laboratory for Green Chemical Process of Ministry of Education, School of Chemical Engineering
and Pharmacy, Wuhan Institute of Technology, Wuhan 430073, China

* Correspondence: lhong@gzhu.edu.cn (H.L.); ljie@wit.edu.cn (J.L.); zqiao@gzhu.edu.cn (Z.Q.);
Tel.: +86-135-6015-8624 (Z.Q.)

† These authors contributed equally to this work.

Table of Contents

Lennard-Jones parameters of MOFs S3

Models of N_2 and O_2 S4

Lennard-Jones parameters and charges of adsorbates S4

Explanation about calculation approach S4

Linear, binomial and trinomial fitting S5

Details of three ML algorithms S6

Details about ML parameters S7

Calculating formulas of the selectivity S8

Predictive importance of six descriptor by three ML algorithms S9

Top ten hMOFs with optimal performance of water harvesting S9

References S9

Table S1. Lennard-Jones parameters of MOFs.[1]

Atom	ε/k_B [K]	σ [Å]	Atom	ε/k_B [K]	σ [Å]	Atom	ε/k_B [K]	σ [Å]
Ac	16.60	3.10	Ge	190.69	3.81	Po	163.52	4.20
Ag	18.11	2.80	Gd	4.53	3.00	Pr	5.03	3.21
Al	254.09	4.01	H	22.14	2.57	Pt	40.25	2.45
Am	7.04	3.01	Hf	36.23	2.80	Pu	8.05	3.05
Ar	93.08	3.45	Hg	193.71	2.41	Ra	203.27	3.28
As	155.47	3.77	Ho	3.52	3.04	Rb	20.13	3.67
At	142.89	4.23	I	170.57	4.01	Re	33.21	2.63
Au	19.62	2.93	In	301.39	3.98	Rh	26.67	2.61
B	90.57	3.64	Ir	36.73	2.53	Rn	124.78	4.25
Ba	183.15	3.30	K	17.61	3.40	Ru	28.18	2.64
Be	42.77	2.45	Kr	110.69	3.69	S	137.86	3.59
Bi	260.63	3.89	La	8.55	3.14	Sb	225.91	3.94
Bk	6.54	2.97	Li	12.58	2.18	Sc	9.56	2.94
Br	126.29	3.73	Lu	20.63	3.24	Se	146.42	3.75
C	52.83	3.43	Lr	5.53	2.88	Si	202.27	3.83
Ca	119.75	3.03	Md	5.53	2.92	Sm	4.03	3.14
Cd	114.72	2.54	Mg	55.85	2.69	Sn	285.28	3.91
Ce	6.54	3.17	Mn	6.54	2.64	Sr	118.24	3.24
Cf	6.54	2.95	Mo	28.18	2.72	Ta	40.75	2.82
Cl	114.21	3.52	N	34.72	3.26	Tb	3.52	3.07
Cm	6.54	2.96	Na	15.09	2.66	Tc	24.15	2.67
Co	7.04	2.56	Ne	21.13	2.66	Te	200.25	3.98
Cr	7.55	2.69	Nb	29.69	2.82	Th	13.08	3.03
Cu	2.52	3.11	Nd	5.03	3.18	Ti	8.55	2.83
Cs	22.64	4.02	No	5.53	2.89	TI	342.14	3.87
Dy	3.52	3.05	Ni	7.55	2.52	Tm	3.02	3.01
Eu	4.03	3.11	Np	9.56	3.05	U	11.07	3.02
Er	3.52	3.02	O	30.19	3.12	V	8.05	2.80
Es	6.04	2.94	Os	18.62	2.78	W	33.71	2.73
F	25.16	3.00	P	153.46	3.69	Xe	167.04	3.92
Fe	6.54	2.59	Pa	11.07	3.05	Y	36.23	2.98
Fm	6.04	2.93	Pb	333.59	3.83	Yb	114.72	2.99
Fr	25.16	4.37	Pd	24.15	2.58	Zn	62.39	2.46
Ga	208.81	3.90	Pm	4.53	3.16	Zr	34.72	2.78

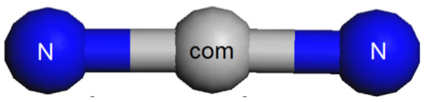

N_2

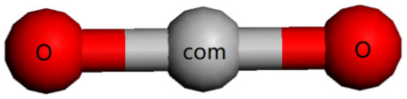

O_2

Figure S1. Models of N_2 and O_2.[2]

Table S2. Lennard-Jones parameters and charges of adsorbates.[3]

Atom	ε/k_B [K]	σ [Å]	Charge (e)
N_N2	36.0	3.31	–0.482
com_N2	0.0	0.00	+0.964
O_O2	49.0	3.02	–0.113
com _O2	0.0	0.00	+0.226
O_H2O	81.9	3.16	0.000
H_H2O	0.0	0.00	+0.524
M_H2O	0.0	0.00	−1.048

Explanation about calculation approach

First, in fact the selectivity under realistic conditions (S_{real}) and infinite dilution (S_0) have some difference. In the previous work[4], the difference between S_{real} and S_0 for the CO_2 mixture were compared, most of S_0 by the calculation of K_i could be good agreement with S_{real} by GCMC. S_0 is slightly lower than S_{real} in region 1, but in region 2 $S_{real} < S_0$. On the whole, S_0 could be used to initially estimate S_{real}. Therefore, S_0 by the calculation of K_i were usually evaluated the adsorption selectivities of MOFs in many previous works.[5]-[7] Secondly, the K_i of H_2O has been applied the screening of hydrophobicity and hydrophobicity for MOFs in the Snurr and co-author's work[8]. In their work, 45 975 hydrophobic MOFs were screened out by K_{H2O}, and the Henry's constants also allowed the efficient calculation of the adsorption selectivity for toxic industrial chemicals and other molecules in competitive adsorption with water. Then, in many other works[9]-[11] the calculation method by K_i also has applied to estimate the hydrophobicity and hydrophobicity for MOFs. Moreover, the target of our work is the capture of H_2O from air, especially for the extremely low concentration of H_2O, such as the desert. In the extreme environment of adsorption, very low content of H_2O can be simply viewed as only one H_2O molecule in the air. Thus, the Henry's constant of water could relatively accurately estimate the adsorption selectivity of H_2O in MOFs under the extreme environment with trace concentration of H_2O.

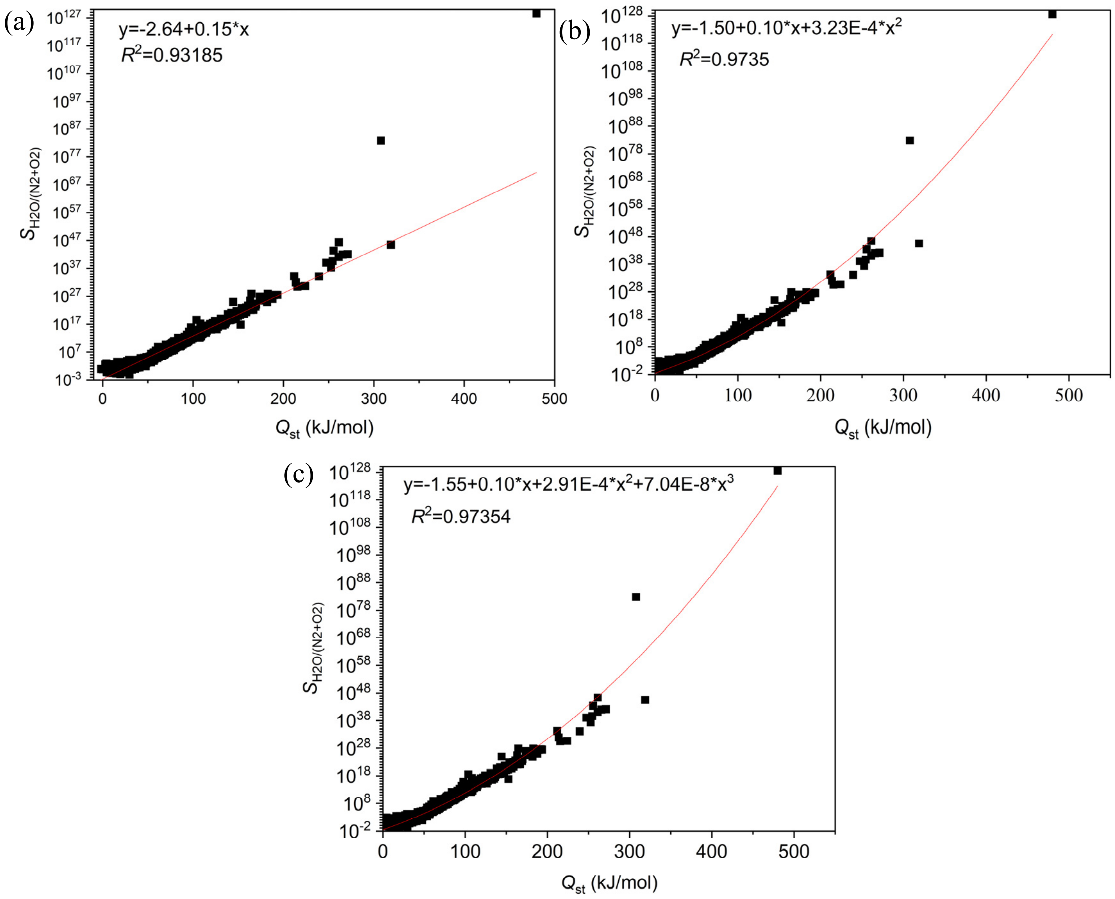

Figure S2. (**a**) Simple linear, (**b**) Binomial and (**c**) Trinomial fitting for $S_{0[H_2O/(N_2+O_2)]}$ to Q_{st}. y represented $\log_{10}S_{H_2O/(N_2+CO_2)}$.

Details of three ML algorithms

Neighbor component analysis

Neighbor component analysis (NCA) is a supervised learning method that can automatically learn a distance metric to compute the distances between samples and make the samples expand as much as possible in the appropriate space, in which dimensionality reduction is achieved. Then a method like k-nearest neighbor algorithm is employed to train the model. Finally, the output variable *i* is predicted, whose value is the average of the several neatest neighbor of *i*. At the meantime, NCA also is a method for non-parametric feature selection, which applies distance metric learned before to characterize the similarity between features, whose purpose is maximizing the accuracy of classification for prediction.

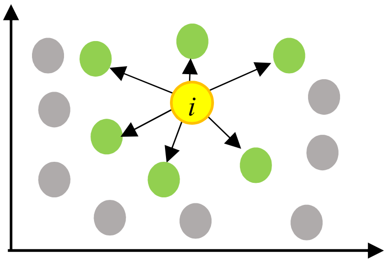

Figure S3. Neighbor component analysis

Gradient boosting regression tree

Gradient boosting machine is one of the ensemble method, the learning procedure consecutively fits new models to provide a more accurate estimate of the response variable. The basic idea of the algorithm is to construct a new basic learner (weak learner) to make it have the greatest correlation with the negative gradient of the loss function and combined with the entire ensemble. There are many types of basic learners for gradient boosting machine, including linear models, smooth models, decision trees, and other models. In our research, our decision tree is used as the base learner, therefore, the algorithm is also called the gradient boosting regression tree (GBRT). The error function we choose is the classic squared error loss (least-squares boosting, LSBoost), and the learning process will result in continuous error fitting. Each of the regression trees learns the conclusions and residuals of all previous trees, and fits a current residual regression tree, as shown in Figure S3.

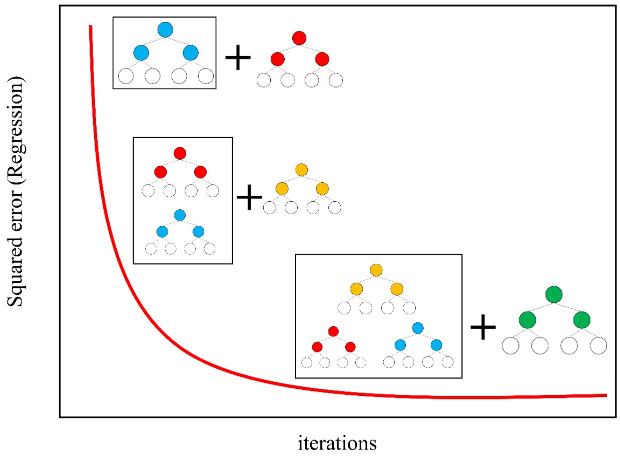

Figure S4. Gradient boosting regression tree

Random forest

Random forest (RF) is consisted by multiple decision trees, which is the improvement and optimization of DT. When training a RF model, multiple samples are randomly selected from independent variable X_i and several features are randomly chosen, and then the features with optimal segmentation were categorized as node for the establishment of DT. Repeating the process N times to build N decision trees, RF selects the average of all trees, whose unbiased estimation makes the model generalization ability stronger than DT.

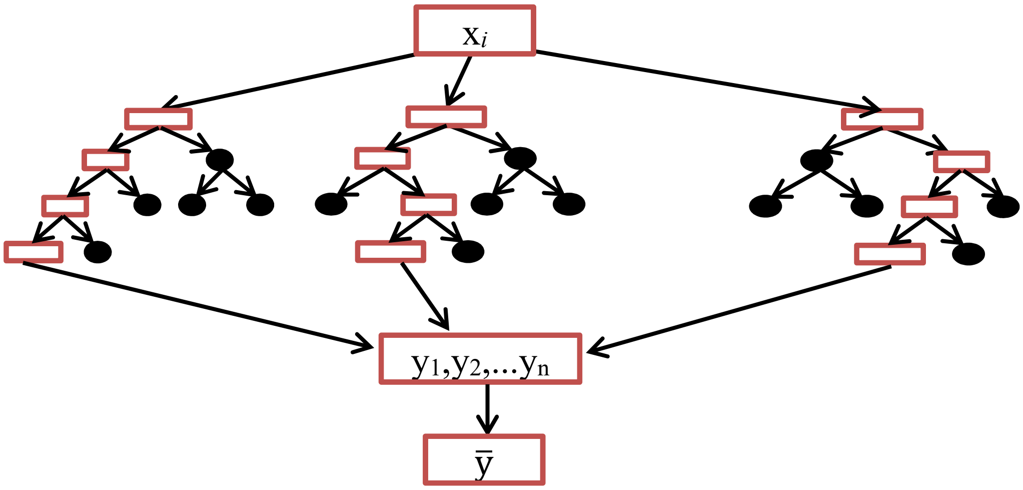

Figure S5. Random forest

Details about ML parameters

The methods for determining the values of parameters are that the 5-fold cross-validation evaluate all possible values of each parameter. And then four ML algorithms programmatically select the optimal parameter values for the final calculation and prediction. The possible values of each parameter are listed in Table S3. The 'Numbers=20' shows that there are evenly spaced 20 points generated in the setting range. For example, in the algorithm of NCA, lambda (regularization parameter to prevent overfitting, value=0-0.05, numbers=20) and IterationLimit (Maximum number of iterations, value=100-2000, numbers=20) will be gridded into 400 (20*20) combinations of parameters. There are also 400 combinations of parameters for GBRT and RF, respectively. Evaluated by the average coefficient of determination(R^2), the cross-validation traverses all combinations of parameters and returns an appropriate model with the best parameter combination. Except for the optimized parameters, the other parameters were the default values. All parameters are listed in Tables S4.

Table S3. Type and the range of key parameters in the optimization

ML	Key parameters	Range of value	Numbers	Best value	Best R^2
GBRT	LearnRate	0.01-0.1	20	0.0479	0.9571
	NumLearningCycle	100-2000	20	200	
NCA	Lambda	0-0.05	20	0.0132	0.9724
	IterationLimit	100-2000	20	300	
RF	MaxNumSplits	10-200	20	160	0.9241
	MinLeafSize	1-20	20	3	

Table S4. Parameters of 4 ML

NCA		RF	
Parameters	Value	Parameters	Value
Method:	'regression'	Method:	regression
FitMethod:	'exact'	NumPredictors:	6
NumPartitions:	10	NumPredictorsToSample:	2
DoFit:	1	MinLeafSize:	3
Lambda:	0.0132	MaxNumSplits:	160
LengthScale:	1	InBagFraction:	1
InitialFeatureWeights:	[6 × 1 double]	SampleWithReplacement:	1
Prior:	'empirical'	ComputeOOBPrediction:	1
Standardize:	1	ComputeOOBPredictorImportance:	1
Verbose:	0	Proximity:	[]
Solver:	'sgd'	NumTrees	200
LossFunction:	'mad'	GBRT	
Epsilon:	0.1579	Parameters	Value
HessianHistorySize:	15	Type:	'regression'
InitialStepSize:	[]	Method:	'LSBoost'
LineSearchMethod:	'weakwolfe'	LearnerTemplates:	'Tree'
MaxLineSearchIterations:	20	NLearn:	200
GradientTolerance:	1.00×10^{-6}	LearnRate:	0.0479
InitialLearningRate:	[]		
MiniBatchSize:	10		
PassLimit:	5		
NumPrint:	10		
NumTuningIterations:	20		
TuningSubsetSize:	100		
IterationLimit:	300		
StepTolerance:	1.00×10^{-6}		
MiniBatchLBFGSIterations:	10		
CacheSize:	1000		
NumObservations:	6013		
NumFeatures:	6		

Calculating formulas of the selectivity

$$S_{(H_2O/(N_2+O_2))} = \frac{2 * K_{H_2O}}{K_{N_2} + K_{O_2}} \qquad S(1)$$

In Formula S (1), The $S_{(CO2/N2+O2)}$ is the adsorption selectivity of H_2O/N_2+O_2, and K_i represents the Henry's constants of component *i* (H_2O, N_2 and O_2).

Table S5. Predictive importance of six descriptor by four ML algorithms

	LCD	ϕ	VSA	PLD	ρ	Q_{st}
NCA	0.0347	1.2790	0.1989	0.0001	0.5190	3.8385
Normalization	0.59%	21.79%	3.39%	0.00%	8.84%	65.39%
GBRT	0.0001	0.0001	0.0000	0.0024	0.0001	0.0786
Normalization	0.16%	0.08%	0.06%	2.89%	0.09%	96.71%
RF	0.7141	0.5131	0.3288	0.4800	0.6695	2.7167
Normalization	13.17%	9.46%	6.06%	8.85%	12.35%	50.10%

Table S6. Details of top ten hMOFs with optimal performance of water harvesting.

**No.**	**ID[a]**	**LCD (nm)**	ϕ	**VSA ($m^2 \cdot cm^{-3}$)**	**PLD (nm)**	ρ **($kg \cdot m^{-3}$)**	Q_{st} **($kJ \cdot mol^{-1}$)**	K_{H2O} **($mol \cdot kg^{-1} \cdot Pa^{-1}$)**	$S_{0[H2O/(N2+O2)]}$
**1**	5005909	1.000	0.49	2174.21	0.711	647.27	1038.53	1.75×10^{193}	6.77×10^{199}
**2**	5078347	0.650	0.37	2147.74	0.556	689.24	459.93	1.61×10^{77}	4.06×10^{82}
**3**	5049453	1.429	0.81	1807.51	1.009	454.90	395.47	4.86×10^{66}	1.12×10^{73}
**4**	25411	0.715	0.70	1867.04	0.652	863.23	356.90	4.00×10^{65}	2.12×10^{72}
**5**	5049861	0.768	0.80	1683.40	0.581	961.38	375.87	1.13×10^{61}	3.02×10^{67}
**6**	10681	0.753	0.47	1372.06	0.510	960.52	271.31	1.06×10^{37}	1.32×10^{43}
**7**	5079423	0.728	0.79	1990.11	0.529	855.88	226.52	2.75×10^{33}	4.12×10^{39}
**8**	10540	0.248	0.44	0.00	0.230	2925.22	244.23	4.19×10^{31}	1.63×10^{38}
**9**	23842	0.629	0.65	1481.92	0.453	939.79	178.43	5.85×10^{24}	1.58×10^{31}
**10**	34632	0.754	0.62	2030.87	0.607	790.91	179.70	4.20×10^{24}	4.51×10^{30}

[a] IDs for hypothetical MOFs.[12]

References

1. Rappe, A.K.; Casewit, C.J.; Colwell, K.S.; Goddard, W.A.; Skiff, W.M. UFF: A Full Periodic Table Force Field for Molecular Mechanics and Molecular Dynamics Simulations. *J. Am. Chem. Soc.* 1992, 114, 10024-10035.
2. Martin, M.G.; Siepmann, J.I. Transferable Potentials for Phase Equilibria. 1. United-Atom Description of n-Alkanes. *J. Phys. Chem. B.* 1998,102, 2569-2577.
3. Shah, M. S.; Tsapatsis, M.; Siepmann, J. I. Development of the Transferable Potentials for Phase Equilibria Model for Hydrogen Sulfide. *J. Phys. Chem. B.* 2015,119, 7041-7052.
4. Qiao, Z.; Zhang, K.; Jiang, J. In silico screening of 4764 computation-ready, experimental metal–organic frameworks for CO2 separation. *J. Mater. Chem. A* 2016, 4, 2105–2114.
5. 1, Watanabe, T.; Sholl, D. S. Accelerating Applications of Metal–Organic Frameworks for Gas Adsorption and Separation by Computational Screening of Materials. *Langmuir* 2012, 28, 40, 14114-14128.
6. 2, Qiao, Z.; Peng, C.; Zhou, J.; Jiang, J. High-throughput computational screening of 137953 metal–organic frameworks for membrane separation of a CO2/N2/CH4 mixture. *J. Mater. Chem. A* 2016, 4, 41, 15904-15912.
7. 3, Deng, X.; Yang, W.; Li, S.; Liang, H.; Shi Z.; Qiao, Z. Large-Scale Screening and Machine

Learning to Predict the Computation-Ready, Experimental Metal-Organic Frameworks for CO2 Capture from Air. *Appl. Sci.* 2020, 10, 569.

8. 4, Moghadam, P. Z.; Fairen-Jimenez, D.; Snurr, R. Q. Efficient identification of hydrophobic MOFs: application in the capture of toxic industrial chemicals. *J. Mater. Chem. A* 2016, 4, 529-536.
9. 5, Yuan, X.; Deng, X.; Cai, C.; Shi, Z.; Liang, H.; Li, S.; Qiao, Z.; Machine Learning and High-Throughput Computational Screening of Hydrophobic Metal-Organic Frameworks for Capture of Formaldehyde from Air. *Green Energy Environ.* 2021, 6, 5, 759-770.
10. 6, Qiao, Z.; Xu, Q.; Cheetham, A. K.; Jiang, J. High-Throughput Computational Screening of Metal–Organic Frameworks for Thiol Capture. *J. Phys. Chem. C* 2017, 121, 40, 22208−22215.
11. 7, Cai, C.; Li, L.; Deng, X.; Li, S.; Liang, H.; Qiao, Z.; Machine Learning and High-throughput Computational Screening of Metal-organic Framework for Separation of Methane/ethane/propane. *Acta Chimica Sinica* 2020, 78, 5, 427-436.
12. Wilmer, C.E.; Leaf, M.; Lee, C.Y.; Farha, O.K.; Hauser, B.G.; Hupp, J.T.; Snurr, R.Q., Large-scale screening of hypothetical metal–organic frameworks. *Nat. Chem.* 2012, *4* (2), 83-89.
